# Weed Classification from Natural Corn Field-Multi-Plant Images Based on Shallow and Deep Learning

**DOI:** 10.3390/s22083021

**Published:** 2022-04-14

**Authors:** Francisco Garibaldi-Márquez, Gerardo Flores, Diego A. Mercado-Ravell, Alfonso Ramírez-Pedraza, Luis M. Valentín-Coronado

**Affiliations:** 1 Centro de Investigaciones en Óptica A.C., Loma del Bosque 115, Leon 37150, Guanajuato, Mexico; franciscogm@cio.mx (F.G.-M.); gflores@cio.mx (G.F.); pedro.ramirez@cio.mx (A.R.-P.); 2 Instituto Nacional de Investigaciones Forestales, Agrícolas y Pecuarias—Campo Experimental Pabellón, Pabellon de Arteaga 20671, Aguascalientes, Mexico; 3 Centro de Investigación en Matemáticas A.C., Lasec y Andador Galileo Galilei, Quantum Ciudad del Conocimiento, Zacatecas 98160, Zacatecas, Mexico; diego.mercado@cimat.mx; 4 Consejo Nacional de Ciencia y Tecnología, Ciudad de Mexico 03940, Mexico

**Keywords:** corn and weed classification, natural environment, multi-plant species, multi-plant image, classic ML, deep learning

## Abstract

Crop and weed discrimination in natural field environments is still challenging for implementing automatic agricultural practices, such as weed control. Some weed control methods have been proposed. However, these methods are still restricted as they are implemented under controlled conditions. The development of a sound weed control system begins by recognizing the crop and the different weed plants presented in the field. In this work, a classification approach of *Zea mays* L. (Crop), narrow-leaf weeds (NLW), and broadleaf weeds (BLW) from multi-plant images are presented. Moreover, a large image dataset was generated. Images were captured in natural field conditions, in different locations, and growing stages of the plants. The extraction of regions of interest (ROI) is carried out employing connected component analysis (CCA), whereas the classification of ROIs is based on Convolutional Neural Networks (CNN) and compared with a shallow learning approach. To measure the classification performance of both methods, accuracy, precision, recall, and F1-score metrics were used. The best alternative for the weed classification task at early stages of growth and in natural corn field environments was the CNN-based approach, as indicated by the 97% accuracy value obtained.

## 1. Introduction

Corn (*Zea mays* L.) is one of the most grown cereals in the world, after rice (*Oryza sativa* L.) and wheat (FAOSTAT data website, http://www.fao.org/faostat/en/#home; accessed on 7 September 2021) (*Triticum aestivum* L.). Furthermore, corn has also become a basic food for some of the poorest countries [1], while for others, it has an important economic impact [2]. For these reasons, genetic enhancements and agronomic practices are still open research areas seeking corn yield improvement, a variable that is directly affected by numerous factors, such as soil fertility, water stress, and weeds.

Weeds are undesirable plants that contribute to 40–60% of yield losses [3], because they compete with the crop for water, nutrients, and sunlight [4]. The current most-worldwide used method to eradicate weeds from crop fields is the chemical method [5], due to its effectiveness and practicality. According to Wang et al. [6], the chemical method is capable of eliminating between 90 to 99% of interrow and intra-row weeds. Nevertheless, this method is causing severe environmental pollution of the soil and groundwater since it is usually sprayed uniformly within crop fields.

Site-Specific Weed Management (SSWM) techniques have been developed, and are still being improved, to mitigate the environmental harm that herbicides cause [7]. These techniques consist of eradicating weeds individually where they lie or in patches, by removing them mechanically, or by thermal, electrical, or chemical means [8,9]. Respecting chemical weed control by SSWM, preliminary studies have stated that it is possible to save from 45 to 66% of herbicides without crop yield decrease, compared to those traditional methods of uniform application [10,11,12]. Recently, Nikolić et al. [13] reported up to 82% of herbicide reduction when SSWM and time-specific weed control (TSWC) were combined.

To implement an automatic weed control technique, the first task to accomplish is the discrimination of weeds from crop plants [14]. Some techniques consist of locating the line of plants [15] to address this issue; then, plants that are outside the line are considered weeds. Other works, such as the one presented by Liu et al. [16], have employed color indices. Nonetheless, they are directly affected by sunlight intensity, as well; they barely give acceptable results when crop and weed share similar green color [14].

Spectral reflectance has also been used, but at early grown stages, weeds and crops have similar reflection [17], making the discrimination complicated. Finally, shape features are the most common methods for the discrimination task, using properties, such as area, perimeter, and major and minor axis of the segmented regions [18,19]; the main drawback with this is that monocot and dicot crops and weeds share similar morphology at early grown stages, making discrimination very difficult.

On the other hand, texture features, which reflect the spatial distribution of pixels, have been reported lately to be efficient for discriminating crops from weeds; since the veins of leaves differ in their texture, and the roughness of their surface also change [20]. The most common texture operators reported for plants discrimination are gray level co-occurrence matrix (GLCM), gray level-gradient co-occurrence matrix (GGCM), and local binary pattern (LBP) [21,22,23,24].

Among these operators, LBP is widely used because it is robust enough to monotonic grey-level transformation, scaling, viewpoint, illumination invariance, and rotation invariance [25]. Furthermore, models based on LBP features have shown breakthrough performance for classifying plants at early grown stages, which is the best stage to obtain the most control of weeds, since their underdeveloped leaf cuticle facilitates easy absorption of active herbicide ingredients [26].

In the work of Le et al. [23], it was reported that the classification performance of the algorithm filtered Local Binary Pattern with contour masks and coefficient k (k-FLBPCM), conjoint with SVM, was better than a deep-learning-based model after being trained on a dataset with mature herbs and tested on a dataset of the early grown stage of plants. Nonetheless, the dataset of Le et al. [23] was acquired under controlled environment conditions.

It seems that for obtaining a considerable acceptable performance when implementing classic machine learning methodologies, after features are extracted, the key is the selection of a correct classifier. The literature reports that random forest [2], K-nearest neighbor (KNN) [27], artificial neural networks (ANN) [13,27,28] and support vector machine (SVM) [29] as the most used crop and weed classifiers. Nonetheless, performance evaluations have shown that SVM is better for this assignment.

For instance, in the work of Bakhshipour and Jafari [18] the authors found that SVM exhibited an overall accuracy of 95% over 92.92% of ANN classifiers when they were trained with shape features of common weeds. The dataset comprises 600 images of five plant species in this previous work. The images were acquired in “real” field conditions; however, the sunlight was obstructed, capturing images at relatively uniform diffuse illumination, allowing easy soil separation from vegetation.

Efforts were made to improve plants’ classification rate and implement Convolutional Neural Network (CNN) models for the same task in common annual crops [30,31]. The motivation arises after the performance that the AlexNet model (a CNN architecture proposed by Alex Krizhevsky et al. [32]) has shown, making it a milestone in classifying images from the ImageNet dataset (a very large collection of human annotated images used for developing computer vision algorithms) in the Large Scale Visual Recognition Challenge in 2012.

What makes CNN models interesting is that they can learn features on their own during the training process, and then, they can discriminate new unseen data at relatively high performance in real close time. Currently, CNN architectures have manifested state-of-the-art performance on classifying plants species. The alexnet model, according to dos Santos Ferreira et al. [33], exhibited an accuracy of 99.5% in a grass-broadleaf dataset. In other work, Ahmad et al. [34] reported an average accuracy of 98.90%, 97.80%, and 96.70% for VGG16, ResNet50, and Inceptionv3, respectively, upon the classification of four weed species.

Most of the reported datasets in the literature for training CNN models have been acquired under controlled light conditions and low background variability, meaning that soil appearance and straws do not change, or even datasets with scarce plant species are used. A considerable quantity of images captured at different scenarios and growing stages of the plants are needed to make it possible to implement CNN in natural field conditions so that their performance does not decay with new data. Additionally, it was found that most CNNs were trained to classify individual plant species, in spite that for weed control, herbicides are selective for NLW and BLW.

As mentioned, shallow and deep learning techniques specialize in classification tasks. Nonetheless, these algorithms should be fed with a single-plant image for its classification. Therefore, in this work, a classification approach of crop plants, common narrow-leaf weeds (NLW), and broadleaf weeds (BLW) from multi-plant images. The principal contribution of this work is the generation of a large dataset of images acquired in a typical cornfield under natural environmental conditions. This dataset contains nine plant species grouped in three classes.

Moreover, another contribution of the work is the comparison of a shallow learning approach, local binary pattern + support vector machine (LBP+SVM), and Convolutional Neural Network (CNN) on the classification of the classes of the built dataset, unlike of those works that classify a small dataset, acquired under controlled environments conditions. In summary, this work proposed a classification system that extracts individual plants from images with multiple plants by using a segmentation algorithm and a Connected Component Analysis algorithm (CCA); converting a simple classification process into a classification vision system for weeds, with applicability in early grown stages of the herbs.

In this way, the remainder of this work is organized as follows; Section 2 details the proposed methods for the dataset generation, image preprocessing, and training of the models. Section 3 shows the results, whereas in Section 4 the discussion of the study is presented. Finally, the conclusions and future work are presented in Section 5.

## 2. Materials and Methods

**Figure 1:**
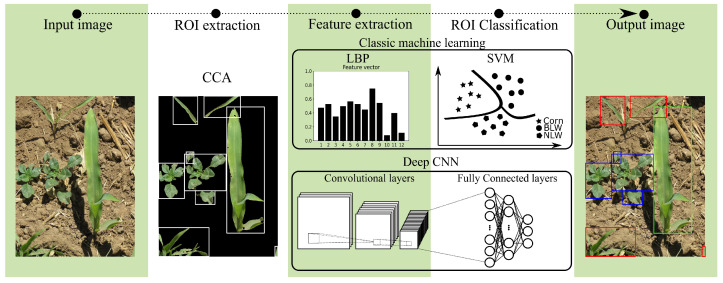
General description of the methodology for weed classification in actual corn fields (green box: Crop, red box: NLW and blue box: BLW).

Classification is the task of predicting the class of given data. However, a large dataset is required to perform this assignment; therefore, in this work, an experimental image dataset was built, and it is detailed in Section 2.1. As Figure 1 shows, the proposed classification process considers five steps. First, images of the field, in natural conditions, are acquired. Then, these images are segmented and enhanced by classic image processing techniques before reaching the second stage. In a second stage, the regions of interest (ROI) in the segmented image are extracted utilizing CCA [35,36].

Afterward, the classification of the objects is done through CNN and classic machine learning methods. The proposed CNN models are based on the well-known VGG16, VGG19 [37], and Xception models [38], which were trained with our dataset. To implement classic machine learning algorithm, primarily, texture features were extracted using the rotation-invariant uniform local binary pattern operator ($LBP_{P,R}^{riu2}$) [22,39]. These features are used to train a Support Vector Machine (SVM) model. At the last stage, the vision system shows the class each of these objects (plants) belongs to.

### 2.1. Dataset Generation and Image Pre-Prossessing

The images were captured in five cornfields located in different regions within Aguascalientes, Mexico. First, a gross dataset integrated of 13,000 images was generated. These images were manually collected in a variety of camera positions in order to have variability in the dataset and avoid further data augmentation.

**Figure 2:**
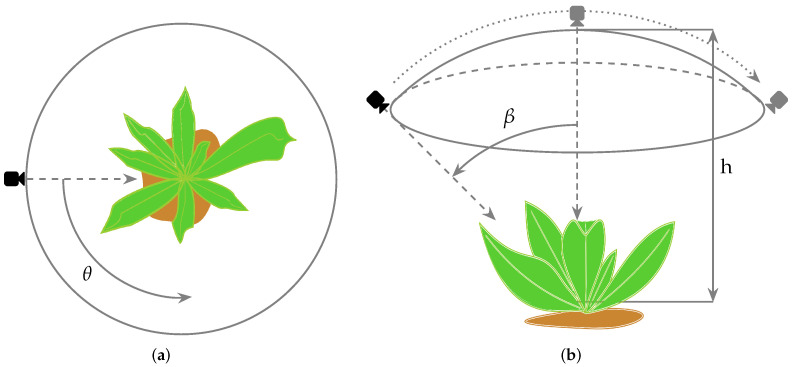
Camera configuration for capturing images. (**a**) top view. (**b**) side view.

Figure 2 shows the locations of the camera that were used to capture the images; where θ∈[0,2π] is the rotational position of the camera respect the target (Figure 2a), and β∈[−π/4,π/4] angle is the lateral orientation of the camera (Figure 2b). When β=0, the top-down camera view is obtained. On the other hand, *h* is the distance between the camera and the target base, which took a maximum value of around 1.50 m and a minimum one necessary for capturing either a corn plant or weed.

**Figure 3:**
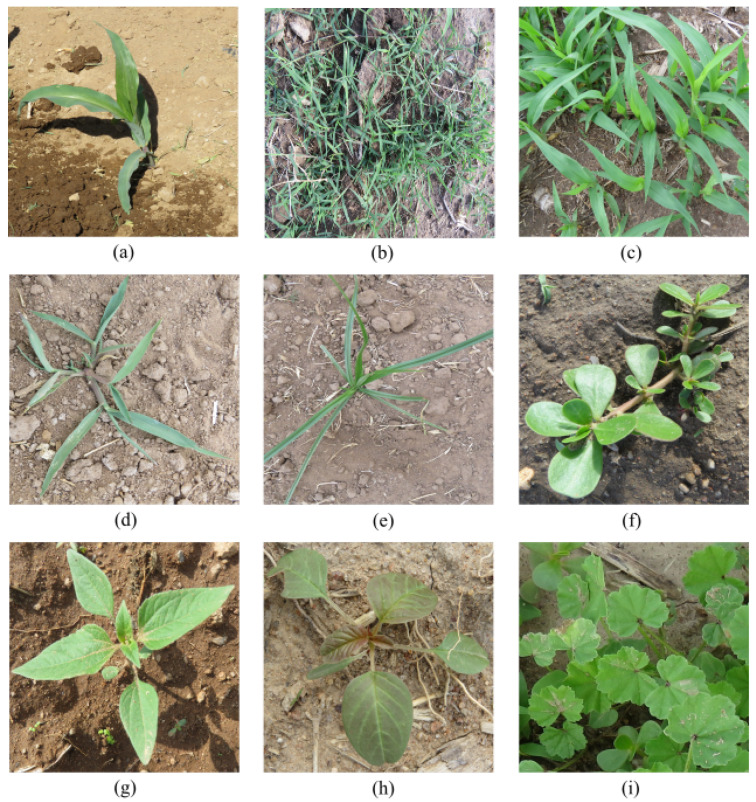
Sample of plants that intagrates the dataset. (**a**) *Zea mays* L. (**b**) *Cynodon dactylon*. (**c**) *Eleusine indica*. (**d**) *Digitaria sanguinalis*. (**e**) *Cyperus esculentus*. (**f**) *Portulaca oleracea*. (**g**) *Tithonia tubaeformis (Jacq.) Cass.* (**h**) *Amarantus spinosus*. (**i**) *Malva parviflora*.

The image acquisition process was performed every five days; as a result, corn and weed plants were of 2–7 leaves in our dataset. This dataset also introduced sunlight variability since the images were collected on sunny and cloudy days. The images were of size 4608×3456 pixels, captured using a Canon PowerShot Sx60HS 16.1-megapixel camera. The gross dataset was integrated by nine plant species, which are presented in Figure 3.

From this gross image dataset, 250 images were left separated for testing the classification system in the natural environment. The remaining images were segmented, followed by their enhancement. Then, CCA was implemented to extract the plants corresponding to the classes Crop, NLW, and BLW to generate a new experimental dataset to train the proposed classification models later. The intuition behind the CCA algorithm is the following.

From a binary image, the first component is initialized with the first white pixel; then, the algorithm scans the image pixel by pixel looking for adjacent pixels and adds these pixels to this component; when no more connected pixels are found, and if there are more pixels, a new component is created. This is repeated until all pixels are assigned to one region. Thus, all pixels assigned to a component are marked with the same unique label [36], allowing to extract the objects by using their labels.

#### 2.1.1. Image Segmentation

Let us define the image $I\in M^{m\times n\times p}$ as the m×n×p hypermatrix, where the ijk-th entry represents the ij-th color pixel for channel *k*, and $M^{m\times n\times p}$ represent all hypermatrices of this type. In this case, the color space of the image is RGB; however, it was reported that this color space is not the best option to separate vegetation from soil [40]; then, a color space transformation from RGB to HSV was implemented.

**Figure 4:**
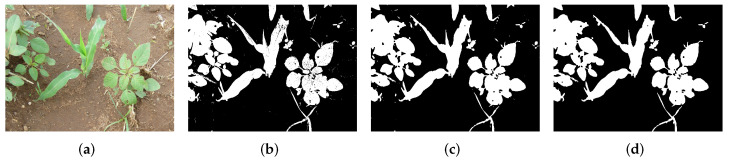
Segmentation stages of images. (**a**) original RGB. (**b**) thresholding output. (**c**) improved image. (**d**) final output mask.

Segmentation in this color space was reported to be upstanding because the color (hue channel) is not correlated with the brightness (value channel), which is better for greenness identification [41]. The resulting image, $I_{hsv}\in M^{m\times n\times p}$, is used to remove the background, this is done using a thresholding function, $B:M^{m\times n\times p}\to M^{m\times n}$, and it is defined by Equation (1).

(1) $$B\left(x,y\right)=\left\{\begin{array}{cc} 255 & \left[H_{l},S_{l},V_{l}\right]-I_{hsv}\left(x,y\right)\leq 0\;\;\mathrm{and}\;\;I_{hsv}\left(x,y\right)-\left[H_{h},S_{h},V_{h}\right]\leq 0\\ 0 & \mathrm{otherwise} \end{array}\right.$$

where $B\left(x,y\right)\in M^{m\times n}$ is the resulting binary image; $I_{hsv}\left(x,y\right)=[I_{hsv}{\left(x,y\right)}_{h},I_{hsv}{\left(x,y\right)}_{s}$, $I_{hsv}{\left(x,y\right)}_{v}]$ is the vector formed by hue, saturation and value channels of the $I_{hsv}$ image; $H_{l},S_{l},V_{l}\in Z^{+}$ and $H_{h},S_{h},V_{h}\in Z^{+}$ are, respectively, the lower and higher values for each of the hue, saturation, and value channels. The thresholding values were tuned manually, and after plenty of iterations on images captured on different light conditions and natural background variability, the threshold values were set as follows: $H_{l}=33$, $H_{h}=95$, $S_{l}=34$, $S_{h}=255$, $V_{l}=60$ and $V_{h}=250$. In Figure 4a a sample of an input image is shown, while in Figure 4b the resulting image of the segmentation step can be seen. It may be noticed that there is some noise distributed within the whole image; hence, image improvement is required.

#### 2.1.2. Image Enhancement

As mentioned, binary images had many holes within the white regions that indicate vegetation. Plenty of small regions appeared where theoretically no vegetation existed, indicating noise. Consequently, the morphological operators opening and closing were executed in the same order to enhance these images. Opening operation smooths the contours of images and eliminates small artifacts. In contrast, the closing operator aids in removing small holes and fills gaps in the contour of regions [42]. At the same time, opening and closing are defined by erosion (Equation (2)) and dilation (Equation (3)) morphological operators [43],

(2) $$A⊖B=\{z|{\left(B\right)}_{z}\subseteq A\neq ⌀\}$$

(3) $$A⊕B=\{z|{\left(\hat{B}\right)}_{z}\cap A\}$$

In the erosion operation, *A* represents all the objects in the binary image, and *B* is the so-called structuring element. Thus, the erosion of *A* by *B* is the set of all points *z*, such that *B* translated by *z* respect to the origin of *B* is contained in *A*. This means that all coincident pixels of *A* and *B* are replaced by pixels of value 0. On the other hand, applying dilation to the binary image A by structuring element B means turning the pixels to value 1 when the center of B matches with the boundary of A. In this way, opening (Equation (4)) comprises an erosion operation followed by the dilation operation. Closing (Equation (5)) operation is defined for a dilation operation followed by an erosion operation.

(4) A∘B=(A⊖B)⊕B

(5) A•B=(A⊕B)⊖B

This work used a structuring element B of size 5×5 for both opening and closing operations. Figure 4c shows the resulting image after applying these morphological operations. However, even though segmentation has been improved, some clusters of pixels still need to be removed. This is achieved through the CCA [35], in such a way that those groups that contain a minimum number of pixels were discarded from the final binary image. Figure 4d shows the segmented image after having carried out the process above.

**Figure 5:**
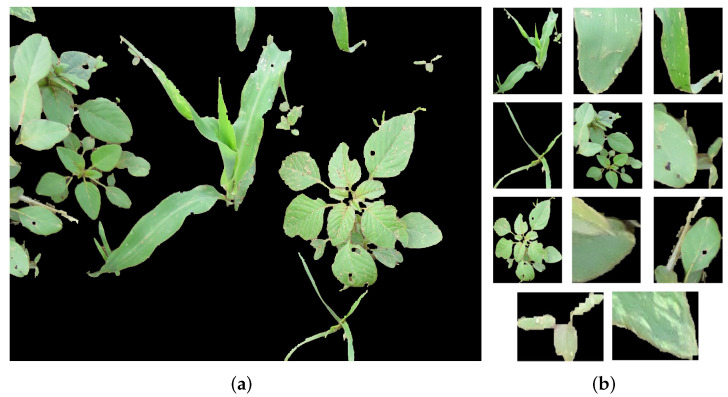
Plant extraction from segmented image. (**a**) Input image. (**b**) Set of different classes.

Therefore, once objects were located in the final binary image, individual objects were extracted from the RGB input image, as shown in Figure 5. Subsequently, the plants were manually classified into the classes Crop, NLW, and BLW to build the experimental dataset for training the models. Table 1 depicts the plant species that integrates this dataset, where, 5080 images integrated each class; furthermore, the plant species inside NLW and BLW were also balanced.

### 2.2. Weed and Crop Classification

According to the two approaches, the built database is used to carry out the classification process. The first one is a classical approach that is based on texture feature extraction from the different plant species that conformed to each class of the experimental dataset (see Section 2.2.1). The second approach is based on the implementation of convolutional neural networks able to characterize and classify the elements of the built dataset (see Section 2.2.2).

#### 2.2.1. Classical Machine Learning Approach

**Figure 6:**
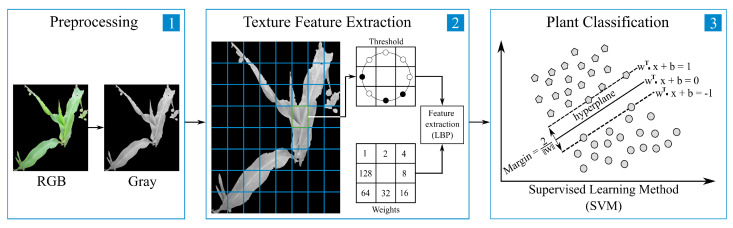
General scheme of the classification approach based on classical machine learning. (**1**) Color space change of the input image. (**2**) Texture feature extraction of the input image through $LBP_{P,R}^{riu2}$. (**3**) Classification of texture features through SVM.

The proposed classical approach is shown in Figure 6 and detailed below. As Figure 6 shows, this approach consists of three stages; in the first one, the RGB image is acquired, and it is pre-processed, making a color space change from RGB to grayscale, while in the second and third stages, the texture feature extraction and the classification are carried out, respectively.

##### Texture Extraction

The rotation-invariant uniform local binary pattern ($LBP_{P,R}^{riu2}$) operator, presented in Ojala et al. [39], was implemented for extracting texture features of the plants under study for their further classification. In addition, as mentioned, the main characteristics of this operator are its monotonic gray-scale transformation, illumination, and rotation invariance [25].

The common LBP algorithm estimates a decimal number, also known as LBP code, for the center pixel $\left(x_{c},y_{c}\right)$ of a 3 × 3 neighbourhood, as follows [39,44],

(6) $$LBP_{P,R}=\sum_{p=0}^{P-1}s\left(g_{p}-g_{c}\right)2^{p}$$

where $g_{c}$ represent the gray value of the center pixel $\left(x_{c},y_{c}\right)$, $g_{p}$ is the gray value of each of the eight neighbors, *P* is the number of pixels in the circular neighbourhood of radius *R*, and s:Z→[0,1] is a function defined as,

(7) $$s\left(x\right)=\left\{\begin{array}{cc} 1, & x\geq 0\\ 0, & x<0 \end{array}\right.,\;x\in Z$$

**Figure 7:**
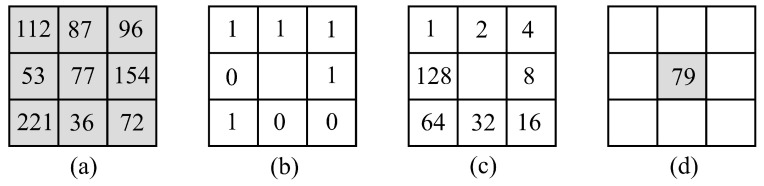
Example of computing the LBP code. (**a**) Fraction of gray-scale image. (**b**) Binary pattern. (**c**) Weights for output patterns. (**d**) LBP code of the central pixel.

Figure 7 describes the process for calculating the LBP code of a 3×3 gray-scale image window. First, the intensity of the center pixel ($g_{c}=77$) is compared with the intensity of each of the eight surrounding pixels ($g_{p}$) (Figure 7a); when the difference value of ($g_{p}-g_{c}$) is greater than 0, it is considered to be 1; otherwise, it is considered to be 0. From this process, an 8-bit binary pattern is obtained, that in this case is 11110010, as shown in Figure 7b. The weights of Figure 7c are calculated by the operation $2^{p}$, wich is a factor of Equation (6). Then, the binary pattern (Figure 7b is element-wise multiplied by the weights (Figure 7c, and the products summed to obtain a LBP code, which in this case is 79. Finally, this LBP code is replaced by the central pixel of the window (Figure 7d).

The LBP algorithm above reflects the texture features by variation of 256 patterns, and the LBP codes are used to construct a histogram of the image to describe the texture features, which is usually normalized for subsequent image classification.

This original LBP operator has a drawback. It fails to capture other outstanding features because only a 3×3 neighborhood is considered and the same number of surrounding pixels. Additionally, not all 256 possible patterns are necessary to extract the most important features [22,25]. Ojala et al. [39] improved the original algorithm and reported this as $LBP_{P,R}^{riu2}$. This sort of pattern has zero or two transitions. When they have zero transition, the pattern is a compound of either zeros or ones, such as 00000000 and 11111111, respectively. A pattern that has two transitions is that which transits from 0 to 1 or from 1 to 0, such as 11001111. In this way, the $LBP_{P,R}^{riu2}$ descriptor is denoted as follows,

(8) $$LBP_{P,R}^{riu2}=\left\{\begin{array}{cc} \sum_{p=0}^{P-1}s\left(g_{p}-g_{c}\right), & \;\mathrm{if}\;\;U(LBP_{P,R})\leq 2\\ P+1, & \mathrm{otherwise} \end{array}\right.$$

where,

(9) $$U\left(LBP_{P,R}\right)=|s\left(g_{P-1}-g_{c}\right)-s\left(g_{0}-g_{c}\right)|+\sum_{p=1}^{P-1}|s\left(g_{p}-g_{c}\right)-s\left(g_{p-1}-g_{c}\right)|$$

##### SVM Classifier Training Stage

Support Vector Machine (SVM), a supervised machine learning algorithm, solves the two-classes classification problem using the following linear model,

(10) $$y\left(x\right)=w^{T}x+b$$

where the parameters w and *b*, the weights and bias, respectively, are calculated from a training dataset of input vectors $x_{1},\ldots ,x_{N}$ with corresponding target values $t_{1},\ldots ,t_{N}$, where $t_{i}\in \left\{-1,1\right\}$, in such a way that new data points *x* are classified according to the sign of y(x). The SVM approaches the classification problem considering the margin concept, which is defined as the smallest distance between the decision boundary and the samples, as shown in Figure 6, stage three.

The margin is calculated by an optimization process of the parameters w and *b* as follows:

(11) $$\underset{w,b}{\mathrm{argmax}}\left\{\frac{1}{\left||w|\right|}\min_{i}\left[t_{i}\left(w^{T}x+b\right)\right]\right\}.$$

To solve this optimization problem, a Lagrange multiplier is needed,

(12) $$L\left(w,b,a\right)=\frac{1}{2}{\left||w|\right|}^{2}-\sum_{i=1}^{N}a_{i}\left\{t_{i}\left(w^{T}x+b\right)-1\right\}$$

where a is a vector of multipliers, whose elements $a_{i}\geq 0$, and *N* are the input vectors. To simplify Equation (12), the derivatives with respect to w and *b* are computed. Next, these derivatives are set equal to zero, resulting,

(13) $$\begin{array}{cc} w= & \sum_{i=1}^{N}a_{i}t_{i}x \end{array}$$

(14) $$\begin{array}{cc} 0= & \sum_{i=1}^{N}a_{i}t_{i}. \end{array}$$

Thus, using these conditions, Equation (12) can be expressed as follows,

(15) $$\tilde{L}\left(a\right)=\sum_{i=1}^{N}a_{i}-\frac{1}{2}\sum_{i=1}^{N}\sum_{j=1}^{N}a_{i}a_{j}t_{i}t_{j}K\left(x_{i},x_{j}\right)$$

with constrains,

(16) $$\begin{array}{cc} a_{i}\geq 0, & \;i=1,\ldots ,N, \end{array}$$

(17) $$\begin{array}{c} \sum_{i=1}^{N}a_{i}t_{i}=0 \end{array}$$

where *K* is a kernel function, which transforms a non-linearly separable space to a linear separable one, and $a_{i}$ is a constant known as the Lagrange multiplier. A more detailed explanation is presented in Bishop [45].

#### 2.2.2. CNN Classification Approach

Convolutional Neural Networks (CNN) are networks that use the convolution operation in each layer to capture spatial and temporal features of the input data. This convolution operation is performed among filters, and the input data, which are in the form of N-dimensional arrays [46]. In contrast to Artificial Neural Networks, CNN significantly reduces the number of learnable parameters [47], which allows them to increase the number of layers [48]. Usually, when the network has more than three layers, they are named deep CNN.

For the reason that VGG16, VGG19 [37] and Xception [38] CNN models have shown excellent performance in the plant classification tasks [23,34,49], in this work, they were evaluated for weed classification in natural field conditions. Another reason for the use of the VGG16 network is that it provides high performance concerning the accuracy, even when it is trained with a dataset with a small number of images [50].

##### VGG Networks

The VGG architectures, also called Visual Geometry Group, are integrated by two and three consecutive convolutional layers followed by a max-pooling layer. The convolutional operations use 3×3 size ReLu kernels. These kernels are smaller than those implied in other CNNs proposed before the epoch they were launched, which usually use 5×5, 7×7 and 11×11 kernel size. In the convolutional layers, the stride is fixed to 1 pixel, and the padding is also of 1 to conserve the spatial resolution of input data. The advantage of the small-size filters is that they are equally efficient extracting features than those large-size-filters; additionally, the number of parameters is reduced, then the computational cost is reduced as well [46]. Respect max-pooling layers of these networks, they use 2×2 size kernels with a stride of 2.

The network has three fully-connected layers (FC) for classification tasks after the convolutional layers. The first two FC layers have 4096 channels with the ReLu activation function. The channels of the last FC layers depend on the number of classes to be classified; for this reason, it comes with a *softmax* activation function.

**Figure 8:**
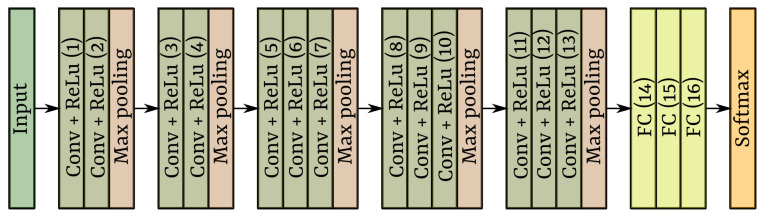
The VGG standard architecture.

The numbers 16 and 19 in VGG16 and VGG19 refer to the number of layers with learnable parameters. Figure 8 shows the VGG16 standard architecture. In the case of VGG19, three consecutive convolutions layers followed by a max-pooling layer are added.

##### Xception Network

Xception is a CNN that was inspired by Depthwise Separable Convolutions (DSC), and Inceptions modules [38]. The DSC, used in previous CNN, such as in Szegedy et al. [51], consists first of Depthwise Convolutions (DC) followed by Pointwise Convolution (PC). DC is a spatial convolution executed separated by the filters over each input data channel, while PC transforms that output data from DC into another channel dimension conserving its spatial size, done through a 1×1 convolution. DSC does not include any activation function among DC and PC. Regarding the Inception module [52], the DSC is implemented in reverse order.

First, PC is performed over the input data, and then DC is executed. Furthermore, in contrast to DSC, an Inception module includes an activation function among PC and DC. The idea of an Inception module is first to seek cross-channel correlations through the 1×1 convolutions and then map the correlations into a small channel dimension. In this way, a common inception module performs three 1×1 convolutional transformations, the PC, and a max-pooling operation in parallel. They are followed by 3×3 and 5×5 convolutions, the DC. The output of these operations is then stacked into a single feature map, equivalent to the dimensions of the channels.

**Figure 9:**
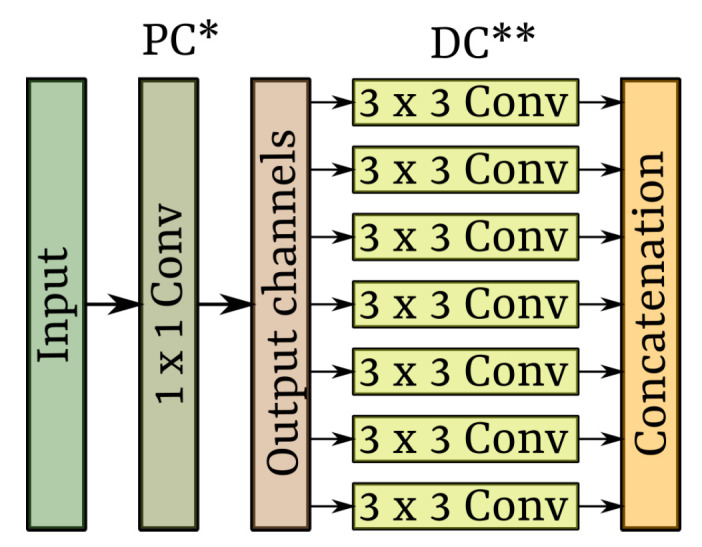
A module of a Xception architecture. * Poinwise convolution; ** Depthwise convolution.

Therefore, an Xception module, similar to the Inception module, first executes PC to map cross-channel correlations and then maps the spatial correlation of each output channel through DC. Nonetheless, the Xception module integrates a single 1×1 convolution as PC. To better visualize this concept, a module of the Xception network is shown in Figure 9. Additionally, similar to DSC, Xception does not include any activation functions among PC and DC. The idea of Xception is to reduce computational cost and preserve the number of parameters, like in Inception.

### 2.3. Performance Evaluation Metrics

To measure the performance of the two proposed approaches, the accuracy, precision, recall, and $F_{1}$-score metrics have been implemented. Accuracy is the ratio between the number of correct predictions and the number of all input samples. This metric works well if the number of samples belonging to each class is equal, which is the case of our dataset. This metric is defined as follows:

(18) $$Accuracy=\frac{TP+TN}{TP+TN+FP+FN}$$

where TP and TN are the true positive and valid negative values, respectively, which, in this context, refers to the plants that were classified correctly into their corresponding class, either positive or negative. FP is the false positive value, which refers to those plants that were classified into a class, but do not belong to it. Lastly, FN is the false negative value, representing the plants belonging to a particular class, but the model does not classify them.

Precision measures the ability of the model to identify targets when it analyzes a certain number of images correctly. It is calculated with the following equation,

(19) $$Precision=\frac{TP}{TP+FP}.$$

On the other hand, Recall indicates the ability of the model to detect targets, and is calculated as follows,

(20) $$Recall=\frac{TP}{TP+FN}$$

Finally, $F_{1}$-score is the harmonic mean of the precision and recall, and is calculated as:

(21) $$F_{1}-\mathrm{score}=2\cdot \frac{Precision\cdot Recall}{Precision+Recall}.$$

Furthermore, the meantime for detecting a single object in an image was registered.

## 3. Results

In this section, the results obtained from applying both classification approaches, SVM- based classical machine learning (Section 2.2.1) and CNN (Section 2.2.2) are presented. It is worth mentioning that both approaches were trained with the built experimental database, which, as already mentioned, consists of 5080 images for each of the three analyzed classes.

The SVM and the CNN models were implemented in a laptop computer with core i7-8550U, Intel UHD Graphics 620, and 16 GB RAM.

### 3.1. Classic Machine Learning

A set of experiments have been carried out to evaluate the performance of the proposed classical machine learning approach in the classification task. As mentioned, the $LBP_{P,R}^{riu2}$ operator was used to extract the texture feature, and the classification was implemented utilizing the SVM. In the case of the LBP operator, three different spatial and angular resolutions (P,R) with values (8,1), (16,2), (24,3) where used. In addition, three different image sizes have also been tested, 256×256, 128×128, and 64×64 pixels, which depending on this size, they were also divided into cells of size 8×8, 16×16, 32×32, 64×64 and 128×128, as Figure 6 illustrates in stage two. The set of combinations are shown in Table 2.

The $LBP_{P,R}^{riu2}$ output feature vector has “P+2” feature patterns. That is, the $LBP_{8,1}^{riu2}$, $LBP_{16,2}^{riu2}$ and $LBP_{24,3}^{riu2}$ operators, have an output vector of 10, 18, and 26 elements, respectively. Therefore, the length of the final concatenated feature vector of each configuration depended on the image size and the images number of cells.

SVM classifiers were trained; therefore, after some iterations, the best kernel function that fitted our data according to the accuracy was linear, meaning that weights were not transformed. The *C* value configuration also started from 1 and gradually increased by a unit. The best accuracy was reached when C=5. The experimental dataset was split into 70% and 20% and 10% for training, validation, and testing. The implementation was done in Python 3.8. The training process was carried out in a laptop computer with core i7-8550U, Intel UHD Graphics 620, and 16 GB of RAM.

**Figure 10:**
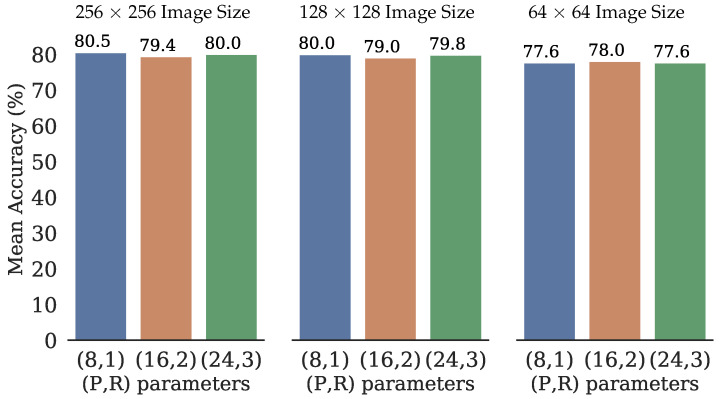
The mean of the Accuracy for each of the (P,R) defined parameters.

The achieved performance of the classifiers is shown in Table 3. Additionally, for each of the $LBP_{P,R}^{riu2}$ texture features, the mean accuracy was calculated. As Figure 10 shows, this means the value is practically consistent among the exact image size for the three $LBP_{P,R}^{riu2}$ operators, existing a difference of less than a unit of magnitude for this variable. Furthermore, the mean accuracy for the image size 256×256 was slightly superior to those other two sizes in each $LBP_{P,R}^{riu2}$ operator. The exact last effect was observed for every one of the metrics precision, recall, and $F_{1}$-score.

Particularly, the best three SVM models were those under the configurations $LBP_{8,1}^{riu2}$/ 256×256/32×32, $LBP_{24,3}^{riu2}$/256×256/32×32, and $LBP_{24,3}^{riu2}$/128×128/32×32, which presented 83.04%, 82.76% and 82.26% accuracy over the test data, correspondingly. This percentage values indicate the percentage of plant species that were classified into their appropriate class. In addition, these models also manifest the same performance behavior for the metrics precision, recall, and $F_{1}$-score. As Table 3 shows, the difference among these variables for every one of these three models is less than one order of magnitude. Regarding test time of these three models, it was less for the model under the configuration $LBP_{24,3}^{riu2}$/128×128/32×32 with 1.89ms of difference respect the model of best accuracy.

### 3.2. CNN Classification

For each of the three CNN models, a transfer learning strategy was implemented; that is, the convolutional layers and their weights tuned in the ImageNet dataset were preserved, and their FC layers were replaced for our proposal. In this regard, the configuration of the FC layers for each model was of two layers. The input layer of 512 channels, followed by a ReLu activation function. For this input layer, the dropout regularization of 0.5 was implemented. The output layer was of three neurons followed by the softmax activation function.

The training process was performed on a desktop computer with Core i7 10700, NVIDIA Quadro P400 graphic processing unit (GPU), and 8 GB of RAM. The implementation was carried out in Python 3.8 and Keras framework with Tensorflow 2.5.0 backend. The experimental dataset was split into 70%, 20%, and 10% for training, validation, and testing, respectively; additionally, the images were resized to 128 × 128 × 3 pixels for the three models. As our dataset comprised three classes, the training was done with *categorical_crossentrpy* loss function, and *Adam optimizer* was used with a learning rate of 0.0001. All models have been trained for 100 epochs with a batch size of 16.

**Figure 11:**
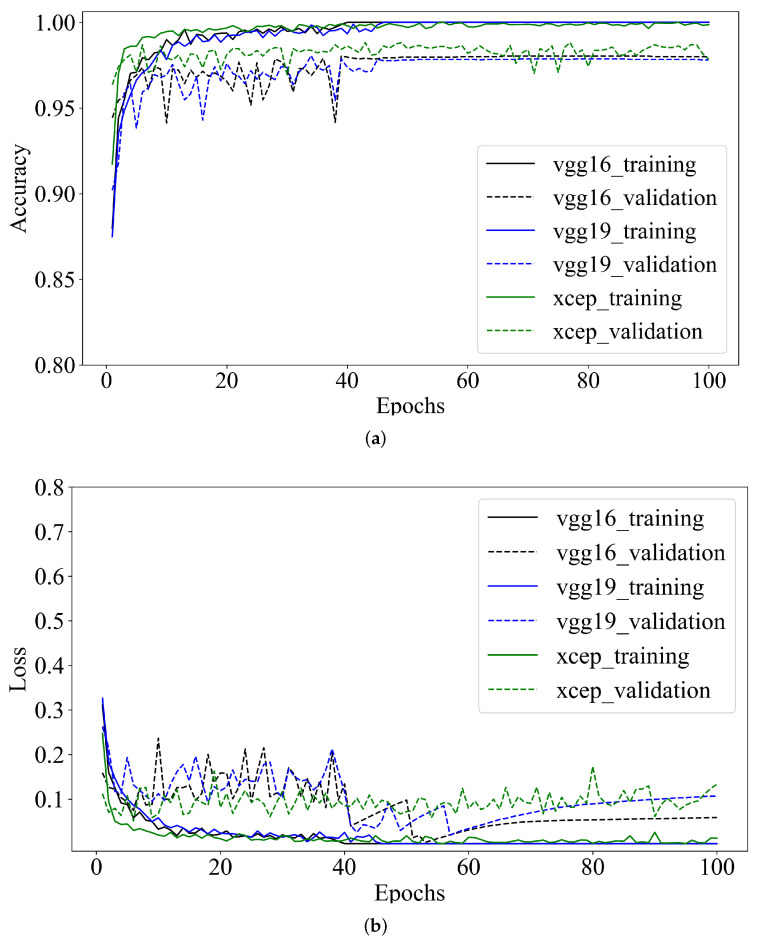
Graphs of behavior during training of VGG16, VGG19 and Xception. (**a**) Accuracy. (**b**) Loss function.

The behavior of the accuracy and the loss function of VGG16, VGG19 and Xception during the training stage are shown in Figure 11.

As Figure 11 shows, from epoch one, the accuracy value increased, and the error value drastically decreased in each of the three models. This behavior is a response to the transfer learning implemented, which usually causes a quick convergence of the models [49], because the weights of the convolutional layers that have already been trained in a distinct dataset are retained, and only the last layers fit the new data.

The accuracy of VGG16 and VGG19 reached its stability for both the training and validation data, from epochs 39 and 45, respectively. In contrast, the accuracy of Xception fluctuated during all the training processes; nevertheless, the amplitude was of less magnitude starting from epoch 48.

Regarding the cost function of each model depicted in Figure 11b, VGG16 was the model that exhibited the slightest error in the validation data from epoch 70, overcoming VGG19 and Xception. Despite that the error of VGG19 had a smooth behavior starting from epoch 58, it showed an incremental tendency until epoch 100, representing overfitting. In the same way, the error of Xception fluctuated during the whole training process; therefore, it can not be considered determinant for this number of epochs for our dataset. The Xception fluctuations among maximum and minimum values of the error during training were also observed by Peteinatos et al. [53]. However, it was superior to the fluctuations of VGG16 and Restnet-50 when trained with twelve species of plants.

The mean performance of VGG16, VGG19, and Xception over the validation data, concerning the accuracy, precision, recall, $F_{1}$-score and time, is provided in Table 4. The mean value of these metrics was in the range 97% and 98%. In general, VGG16 was the best model, whose accuracy was 97.83%. The same order of performance was exhibited for the metrics precision, recall, and $F_{1}$-score compared to VGG19 and Xception, wherein in all the cases, the difference was also less than one order of magnitude. The best test time was reached by Xception, which was 50.18 ms faster than VGG16.

### 3.3. Comparison of Classic Machine Learning and CNN

In this section, a comparative analysis of the best three classic machine learning models and the three CNN models is presented. Let SVM${}_{A}$ be the model trained with $LBP_{8,1}^{riu2}$/256×256/32×32, SVM${}_{B}$ the model trained with $LBP_{24,3}^{riu2}$/256×256/32×32, and SVM${}_{C}$ the model trained with $LBP_{24,3}^{riu2}$/128×128/32×32.

**Figure 12:**
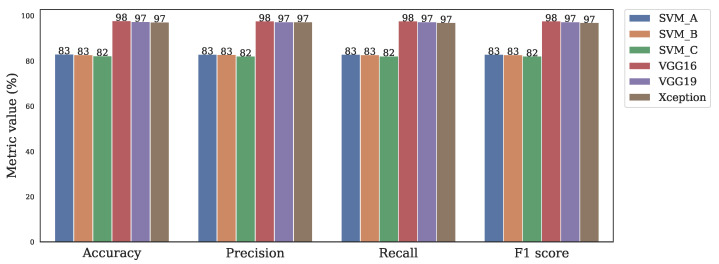
Comparison of classification approaches.

Figure 12 shows the comparison between the three best classic machine learning approaches and the three CNN models. It can be appreciated that the mean performance of the CNN models outreached the SVM models. For example, the mean accuracy of VGG16, which was the best CNN model, overcame in 14.79% the SVM${}_{A}$, which was as well the best classic machine learning model. Additionally, VGG16 was 1.11x faster than $SVM_{A}$ for analyzing an image.

**Figure 13:**
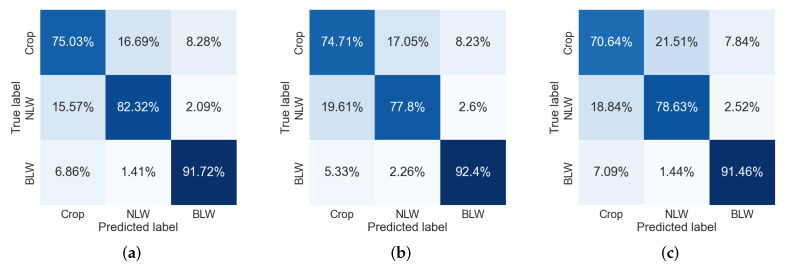
Confusion matrices of the three SVM models. (**a**) SVM${}_{A}$. (**b**) SVM${}_{B}$. (**c**) SVM${}_{C}$.

Furthermore, confusion matrices have been created to evaluate the performance of each model. Figure 13 shows the three confusion matrices for each of the SVM models. It can be seen that the maximum rate reached was 92.4% for BLW by SVM${}_{B}$ (Figure 13b). Nevertheless, it can also be observed that all the models are confused when trying to classify both classes “Crop” and “NLW”, assigning instances of the class “Crop” to “NLW” and vice versa. In the best case there is around 15% of confusion, while for the worst case a misclassification of up to 21% is presented.

The best-identified class was BLW, then NLW, and the worst one was Crop for each model. The identification of BLW reached 92.4% for SVM${}_{B}$, as it was mentioned. Concerning NLW and Crop, both classes were best identified by SVM${}_{A}$, with 82.32% and 75.03%, respectively. A possible explanation for why the models confuse classes “Crop” and “NLW” is that both classes belong to the monocot species and share many texture features.

**Figure 14:**
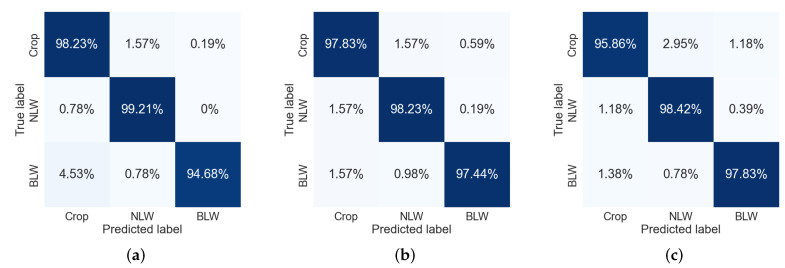
Confusion matrices of the three CNN models. (**a**) VGG16. (**b**) VGG19. (**c**) Xception.

On the other hand, Figure 14 shows the confusion matrices of the CNN models. In this case, Crop and NLW were better classified by the VGG19 model, reaching 98.23% and 99.21%, respectively, while Xception 97.83% best classified BLW. The VGG16 CNN model, with better mean accuracy, exhibited a more uniform classification between classes; the maximum difference was 0.79% among NLW and BLW. Moreover, in agreement with SVM models, they also confused Crop with NLW and vice versa in more degree than with BLW.

Xception misclassified 2.95% of Crop into NLW versus 1.57% by VGG16 and VGG19. NLW was classified as Crop in 1.57% by VGG16, the most misclassification of the class by the models. As well, predominantly, BLW was misclassified as Crop than NLW by the three models. According to the results, it is clear enough that the three CNN models outperformed the obtained results of the three SVM models.

## 4. Discussion

A vast quantity of images captured at different scenarios and growing stages of the plants are needed to implement a classification vision system in real field conditions so that its performance does not decay when the system is fed with unseen data. In this way, our dataset is integrated by images of eight common species of weeds and the corn crop. Images were captured in different corn field locations and in different grown stages of the plants. The sunlight variability and the natural background in each image were also introduced. Therefore, our dataset could give new models the potential to be transferred on natural corn field applications once they are trained on it.

In the same way, the reality in the control of weeds, when a crop is already established, is the use of selective herbicides for NLW or BLW [54]. This could be a drawback for those models trained to classify single plant species. Therefore, we consider that our best SVM model gave an acceptable accuracy (83.04%), considering the variability in our dataset and because the plant species were grouped into the classes crop, NLW, and BLW. In this case, the SVM model had to learn the complexity of the features that come from each of the distinct plant species and relate them into a single class, making the classification a complex task.

Janahiraman et al. [55] also evaluated the performance of the models $LBP_{8,1}^{riu2}/SVM$ and $LBP_{16,2}^{riu2}/SVM$ over BLW classification from the Flavia dataset [56], obtaining a mean accuracy of 64.22% and 75.49%, respectively. When those same models were evaluated in the Swedich dataset (https://www.cvl.isy.liu.se/en/research/datasets/swedish-leaf/; accessed on 29 October 2021), which is a BLW dataset, the mean accuracy was 78.44% and 85.56% for $LBP_{8,1}^{riu2}/SVM$ and $LBP_{16,2}^{riu2}/SVM$, correspondingly. However, the two datasets were acquired under controlled light conditions, and the images present uniform background.

On the other hand, in the work presented in Chen et al. [24], the authors reported a mean accuracy of 90.60% for an SVM model that was trained with texture features of corn and weeds under the configuration $LBP_{8,1}^{riu2}/256\times 256/64\times 64$. Even though the dataset of Chen et al. [24] was generated in actual field environments, it was integrated by 2000 images and contains the classes crop and weeds. The weed class is integrated into two NLW and two BLW plant species. They were making the model with less chance to generalize to unseen species of plants.

Among CNN models, the best was the VGG16, which reached a mean accuracy of 97.83% on classifying Crop, NLW, and BLW in natural field environments, when four plant species integrate NLW and BLW. Most of the reported works in the literature have been focused on the classification of individual species of plants. When individual species of plants are classified, superior performances to 97% have been reported in the literature for VGG16 and VGG19.

However, the number of species worked with have been only four [23,34] or five [57]; in addition, this mean performance has been reached with a reduced number of images for each plant species for training the models. In contrast, when multiple species have been used for training CNN models, the performance typically decays [58,59]. There are scarcely reported works in the literature when weeds have been integrated into classes NLW and BLW.

Yu et al. [60] reported that VGG16 reached a mean accuracy of 99% for classifying more than five broadleaf weed species integrated into a single class over Dormant Bermuda grass. However, the environment was uniform due to its appearance compared to the BLW appearance, triggering an easy weeds differentiation. On the contrary, in the work of dos Santos Ferreira et al. [61] a mean accuracy of 83.4% was reported for VGG16 when it was trained with plants of soja, soil, and grass-broadleaf weeds; this last class integrated with multiple plant species. This makes our work interesting since scarce information was found when CNN is trained with classes Crop, NLW, and BLW into real environments of cornfields.

Using a CNN-based approach has shown to be better than the classical machine learning approach, as in all scenarios has shown better results. Then, for the weed classification task at early stages of growth and in natural environments, like the one presented in this work, an accuracy value of 97.50% (on average) indicates that the CNN-based approach is the best alternative to perform this assignment.

The classification vision system extracts the multiple plants from a simple image and then classifies them into their corresponding class. The classification process in this work was evaluated on 250 images, as already mentioned.

Other fields of science, such as medicine or engineering, may take advantage of systems like the one presented in this work since it can help to improve the decisions-making process by providing very helpful information. Some intances of application are those for desease diagnosis of plants [62], disease diagnosis of humans [63], and fault diagnosis of engineering elements like the one presented in Glowacz [64]. Therefore, the study of intelligent classifiers is still an open research area.

## 5. Conclusions

This work proposes a classification vision system to classify individual plants from multi-plant images captured in real cornfield environments. Therefore, a dataset of 15,240 images that contains nine plant species, grouped into the classes Crop, NLW, and BLW, was generated. Images were captured under these real cornfield environments, and plants were of different growth stages. The classification of the plants of the dataset was carried out by a classical approach and by CNN.

For the classical approach, the $LBP_{P,R}^{riu2}$ operator was used to extract texture features for the three spatial and angular resolution (P,R) equals (8,1), (16,2) and (24,3). The image sizes 256×256, 128×128 and 64×64, and the cell sizes of the images 8×8, 16×16, 32×32, 64×64 and 128×128 were also tested into this approach. The classification of the resulting texture feature dataframes has been implementing utilizing the SVM. The best three SVM models were those under the configurations $LBP_{8,1}^{riu2}$/ 256×256/32×32, $LBP_{24,3}^{riu2}$/256×256/32×32, and $LBP_{24,3}^{riu2}$/128×128/32×32, which reached correspondingly 83.04%, 82.76% and 82.26% of accuracy.

The evaluated CNN were VGG16, VGG19 and Xception for the classification of the herbs of our dataset. In this regard, the accuracy of VGG16, VGG19 and Xcption was 97.93%, 97.44% and 97.24%, respectively.

The best three classic machine learning classified better the class BLW. In contrast, the three deep CNN models classified slightly better the class NLW. In general, the performance of the best classic model and the best CNN model was acceptable compared to those models trained under datasets captured in natural environments. This study also indicates that CNN is better for classification tasks in these actual field conditions when crop and weed are in early grown stages.

The extraction algorithm of ROI by using the segmentation of the soil from the green regions works well when the plants are at early growing stages; this is when occlusion and overlap do not exist in the field. However, one ROI could be integrated by more than a class when these parameters are present. Therefore, future research will be conducted for our research team to conjoint the use of standard classification CNN models with automatic ROI detection algorithms. Also, images of the dataset are being annotated at a pixel level.

## Figures and Tables

**Table 1:** Plant species that integrate the experimental dataset.

Class	Scientific Name	Common Name	Number of Instances
Crop	*Zea mays* L.	Corn	5080
NLW	*Cynodon dactylon*	Bermudagrass	5080
	*Eleusine indica*	Goosegrass	
	*Digitaria sanguinalis*	Large crabgrass	
	*Cyperus esculentus*	Yellow Nutsedge	
BLW	*Portulaca oleracea*	Common Purslane	5080
	*Tithonia tubaeformis (Jacq.) Cass.*	–	
	*Amaranthus spinosus*	Spiny Amaranth	
	*Malva parviflora*	Little Mallow	

**Table 2:** Configuration of experimental dataset for texture feature extraction.

${\mathrm{LBP}}_{P,R}^{\mathrm{riu}2}$	Image Size	Cell Size				
		8 × 8	16 × 16	32 × 32	64 × 64	128 × 128
P = 8, R = 1	256 × 256	√	√	√	√	√
	128 × 128	√	√	√	√	
	64 × 64	√	√	√		
P = 16, R = 2	256 × 256	√	√	√	√	√
	128 × 128	√	√	√	√	
	64 × 64	√	√	√		
P = 24, R = 3	256 × 256	√	√	√	√	√
	128 × 128	√	√	√	√	
	64 × 64	√	√	√		

**Table 3:** Performance of SVM models trained with $LBP_{P,R}^{riu2}$ texture features.

(P,R)	Metrics	256 × 256 Image					128 × 128 Image				64 × 64 Image		
		Cell Size					Cell Size				Cell Size		
		8 × 8	16 × 16	32 × 32	64 × 64	128 × 128	8 × 8	16 × 16	32 × 32	64 × 64	8 × 8	16 × 16	32 × 32
(8,1)	Accuracy (%)	79.39	79.39	**83.04**	81.50	79.28	77.34	80.31	81.82	80.35	77.60	78.32	77.01
	Precision (%)	79.82	79.73	82.94	81.44	78.95	77.50	80.23	81.65	80.22	77.64	78.22	76.74
	Recall (%)	79.40	79.41	82.90	81.59	79.16	77.30	80.22	81.72	80.31	77.75	78.27	76.93
	$F_{1}$ score (%)	79.54	79.54	82.91	81.50	79.03	77.39	80.21	81.65	80.21	77.68	78.24	76.81
	Test time (ms)	407.65	235.57	212.07	206.46	205.03	227.98	211.58	204.57	200.28	205.70	199.77	198.25
(16,2)	Accuracy (%)	79.72	79.3	81.36	79.96	76.5	77.54	80.53	79.65	78.45	77.08	80.31	76.73
	Precision (%)	80.02	79.96	81.23	79.69	76.03	77.69	80.70	79.72	78.52	77.07	80.27	76.71
	Recall (%)	79.75	79.40	81.34	79.82	76.37	77.29	80.64	79.88	78.63	77.14	80.33	76.05
	$F_{1}$ score (%)	79.87	79.57	81.27	79.74	76.13	77.42	80.67	79.73	78.58	77.10	80.30	76.86
	Test time (ms)	439.53	250.37	221.68	215.32	213.08	241.71	210.24	202.42	202.20	207.66	200.71	199.02
(24,3)	Accuracy (%)	77.07	80.77	**82.76**	81.27	78.12	77.93	81.34	**82.26**	77.62	76.99	78.78	77.16
	Precision (%)	77.56	81.20	82.80	81.10	77.69	78.17	81.40	82.13	77.60	77.30	78.73	77.79
	Recall (%)	77.08	80.82	82.77	81.20	77.90	77.90	81.38	82.18	77.76	77.02	78.93	77.10
	$F_{1}$ score (%)	77.19	80.97	82.78	81.14	77.77	78.01	81.38	82.14	77.67	77.11	78.76	76.90
	Test time (ms)	461.86	263.72	228.92	220.50	217.75	257.94	217.10	210.18	203.83	220.46	205.14	200.28

**Table 4:** Performance of CNN models.

Model	Accuracy (%)	Precision (%)	Recall (%)	$F_{1}$ Score (%)	Test Time (ms)
VGG16	97.83	97.67	97.67	97.67	194.56
VGG19	97.44	97.33	97.33	97.33	226.96
Xception	97.24	97.33	97.00	97.00	144.38

## Data Availability

The dataset for this study is being annotated at the pixel level. Therefore, it is not available at present.
